# Therapeutic Effect on Swallowing Function and on Hydration Status of a New Liquid Gum-Based Thickener in Independently-Living Older Patients with Oropharyngeal Dysphagia

**DOI:** 10.3390/nu15214621

**Published:** 2023-10-31

**Authors:** Noemí Tomsen, Mireia Bolívar-Prados, Omar Ortega, Pere Clavé

**Affiliations:** 1 Gastrointestinal Physiology Lab, Hospital de Mataró, Consorci Sanitari del Maresme, 08304 Mataró, Spain; 2 Centro de Investigación Biomédica en Red en Enfermedades Hepáticas y Digestivas, Instituto de Salud Carlos III, 28029 Madrid, Spain

**Keywords:** swallowing disorders, thickening product, rheology, therapeutic uses, hydration

## Abstract

ThickenUp^®^ Gel Express (TUGE) is a new, xanthan- and acacia-gum-based, liquid, thickening product. In independently living older adults with oropharyngeal dysphagia (OD), we assessed: (1) the rheological properties of TUGE; (2) its therapeutic effect at four viscosity levels (achieved by 5 g, 10 g, 20 g and 30 g of TUGE in water + Omnipaque X-ray contrast) versus thin liquid; and (3) the effect on hydration status and gastrointestinal tolerance after fourteen days. Shear viscosity of TUGE was measured in SI units (mPa·s at 50 s^−1^). The Penetration Aspiration Scale (PAS) score and the swallow response at each viscosity level was assessed with videofluoroscopy (VFS), and in the 14-day study we assessed fluid intake, hydration, and tolerance. Thickened fluids with TUGE were unaffected (−0.3%) by α-salivary amylase (α-SA). The shear viscosity values with VFS were 49.41 ± 2.38, 154.83 ± 10.22, 439.33 ± 11.72 and 672.5 ± 35.62 mPa·s. We studied 60 independently living adults (70 ± 11.4 years) with mild OD (PAS 4.1 ± 2.2, 25% aspirations). TUGE caused a shear-viscosity-dependent improvement in PAS at 150–670 mPa·s and in safety of swallow, slightly increased oral residue, did not affect pharyngeal residue and reduced time to laryngeal vestibule closure (−27%) at 670 mPa·s. Fluid intake with TUGE (1488 mL/day) was well tolerated, and hydration status improved. In conclusion, TUGE was unaffected by α-SA and strongly improved safety of swallow in a viscosity-dependent manner without affecting pharyngeal residue. Fourteen-day treatment of thickened fluids with TUGE is safe and well tolerated and improves hydration status in older adults with dysphagia.

## 1. Introduction

Oropharyngeal dysphagia (OD) is a symptom of a swallow dysfunction that provokes difficulty or inability to form or move the alimentary bolus safely from the mouth to the esophagus and includes oropharyngeal aspirations (entry of oral content from the oropharynx into the trachea or the lungs) and choking (the subsequent mechanical obstruction of pulmonary air flow) [1,2]. Swallowing disorders (dysphagia) have been recognized by the World Health Organization as a medical disability associated with increased morbidity, mortality and costs and included in the International Classification of Diseases (ICD-11: MD93) [1,3].

OD is a highly prevalent condition in the growing older population, with the highest prevalence in frail older patients (>60%) or those affected by neurological conditions such as stroke (>50%) or neurodegenerative diseases (>80%) [1,2,4]. Aging of the society is a known phenomenon that will increase the occurrence of highly prevalent geriatric syndromes such as OD in the world population [2]. OD and its complications are related to impaired clinical outcomes such as hospital readmissions and mortality, impaired quality of life for patients and their family and caregivers and increased healthcare costs [2,5,6,7]. OD is associated with the development of respiratory infections, including aspiration pneumonia, due to impaired swallowing safety, and with malnutrition (MN) and dehydration (DH) due to impaired swallowing efficacy [2]. Since MN is accompanied by loss of muscle mass and function, also affecting masticatory and swallowing muscles, OD is self-reinforcing and may trigger the frailty process in older persons. DH may contribute to mental confusion, vertigo, physical weakness, and fatigue, and thus also promote frailty [8,9]. Described prevalence of MN in OD patients ranges from 20% to 45%, depending on patient phenotype, and can exceed that in specific situations [2]. DH is also a prevalent complication of OD; however, it is highly underdiagnosed. DH has been found in 19% to 100% of patients with OD, and its prevalence increases with OD severity [10,11,12,13].

The well-established treatment of OD is mainly based on compensatory measures such as the use of specific products to compensate for the biomechanical alterations that these patients have and the use of postures and maneuvers with the same purpose (classical treatment), but new neurorehabilitation treatments are now emerging [1,14]. Regarding specific products for OD (thickening products-TP-), these change the viscosity of fluids and the texture of solids to reduce the risk of aspiration and choking [2,15,16,17]. Two of the main properties to take into account for these thickening products are: (1) their resistance to α-salivary amylase enzyme during the oral phase, and (2) the shear-thinning effect caused by bolus flow during the pharyngeal phase. α-salivary amylase can break O-glycosidic bonds, highly prevalent in starch-based thickeners, which results in a marked decrease in fluid viscosity, putting the patient at risk of aspiration [18]. On the other hand, shear rate, defined as the rate at which a fluid is sheared during flow due to bolus velocity during the swallowing process ranging from 1 to 1000 s^−1^, decreases viscosity as its own value and bolus velocity increases, an effect called shear thinning [18,19,20].

The type and duration of nutrition interventions depend on type and extent of the swallowing disorder, nutritional status, and comorbidity, and should be individualized after a detailed assessment of the patient’s current state [2]. There are high-quality studies that support the beneficial effect of thickened fluids in maintaining hydration status in these patients [10,21,22]. However, the effects of TP on the pathophysiology of impaired swallow responses are not fully understood, there is no agreement on the degree of bolus thickening needed, and manufacturers’ labeling is very heterogeneous and even inaccurate [23]. The European Society for Swallowing Disorders (ESSD) stated that “there is evidence for increasing viscosity to reduce the risk of airway invasion and that fluid thickening is a valid management strategy for OD. However, new TP should be developed to avoid the negative effects of increasing viscosity on residue, palatability, and treatment compliance and they have to be easy-to-use”. New clinical trials for each available product should establish the optimal viscosity level for each phenotype of patient with OD and standardize the terminology used and measure viscosity in the International System of Units (SI) [23]. Previous studies of ours and by others have evaluated the therapeutic effect of several TP of a new generation of gum-based thickeners and found they improved swallowing safety and efficacy, as well as compliance, acceptability, and gastrointestinal (GI) tolerance compared to thin liquids [24,25,26].

The aim of this study was to assess the rheological properties (amylase resistance, shear thinning), and the therapeutic effect on swallowing function of a new xanthan- and acacia-gum-based liquid thickening product (TP) with a dosing dispenser (ThickenUp^®^ Gel Express (TUGE), Nestlé Health Science, Vevey, Switzerland) in older patients with OD. We used videofluoroscopy (VFS) to compare fluids thickened with TUGE at increasing viscosities with thin liquid. We also evaluated the effect of these thickened fluids on hydration status (analytical parameters, BIA and fluid intake) and product acceptability and GI tolerance in a subgroup of these patients in a 14-day intervention study.

## 2. Materials and Methods

### 2.1. Study Product and Sample Preparation

The thickening product (TP) used was ThickenUp^®^ Gel Express (Nestlé Health Science, Vevey, Switzerland) composed of arabic instant gum and xanthan gum and has a dosing dispenser. The levels of viscosities in the study were obtained by adding 1 (5 g), 2 (10 g), 4 (20 g) or 6 (30 g) pushes on the TUGE dispenser into 150 mL of Vittel water (Nestlé, Vevey, Switzerland) for volume–viscosity swallowing test (V-VST) and hydration studies, or into 75 mL of Vittel water plus 101.2 g of Omnipaque X-ray contrast (GE HealthCare, Illinois, USA) for VFS studies. A minimum duration time of 15 min between preparation and administration was established. The mixtures were stirred for 10 s clockwise, 10 s anticlockwise, and 10 s clockwise (30 s) at approximately 20 revolutions/10 s. The amount of sodium per push is 25 mg.

### 2.2. Rheological Characterization

The shear viscosity value of each viscosity level used in the clinical studies was assessed in SI units (mPa·s) using a Haake Viscotester™ 550 with RheoWin Software (Thermo Fisher Scientific, Waltham, MA, USA). An MV1 rotor was used for fluids with a viscosity less than 300 mPa·s, and an SV1 rotor for fluids more than 300 mPa·s; 10 mL of the thickened fluid was placed in the gap of the rotor and measured in a shear rate frame from 1 to 1000 s^−1^ at 25 °C. Viscosity at 50 s^−1^ and at 300 s^−1^ was interpolated from the regression line.

The rheological characterizations included, (a) Apparent viscosity: corresponding to the basal viscosity of the thickened fluids at 50 s^−1^ of the four different doses of thickener; (b) Shear rate effect: decrease caused by shear rate (shear thinning) calculated according to the viscosity differences at 300 s^−1^, taking pre-oral incubation viscosity at 50 s^−1^ as reference value; (c) Alpha-amylase effect: this effect was determined after an oral incubation of the investigation products and analyzed following the same methodology described above. To perform this procedure, 3 healthy volunteers held 15 mL of the study product in the mouth for 30 s, then spat it into a glass. Viscosity decrease caused by alpha-amylase was calculated according to the differences in viscosity, taking pre-oral incubation at 50 s^−1^ viscosity as reference value [18,27], and (d) X-ray contrast effect: the effect of the X-ray contrast, Omnipaque, was calculated according to the differences in viscosity between the thickened fluid with water plus contrast.

### 2.3. Clinical Study Design

This was a single-center, interventional, prospective, open-label study on patients with OD (NCT04741581). The study was divided into two parts.

**Figure 1:**
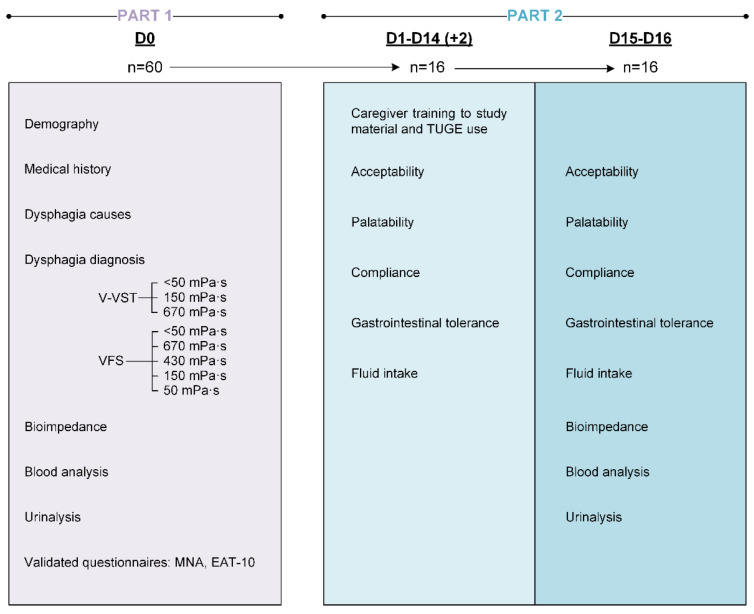
Overall study design: Part 1 (VFS study) and 2 (14-day hydration study). D0: day 0 (baseline visit); D1-D14: day 1 to day 14 (intervention period); D15-D16: day 15 to day 16 (follow-up visit); V-VST: volume–viscosity swallowing test; VFS: videofluoroscopy; MNA^®^: Mini Nutritional Assessment; EAT-10: Eating Assessment Tool-10: TUGE: ThickenUp^®^ Gel Express.

Part 1 was performed in one visit. First, patients were clinically assessed for OD with the Volume–Viscosity Swallowing Test (V-VST) [28] and, if positive for OD (signs of impaired efficacy and/or safety of swallow), they underwent VFS. During the VFS procedure, we assessed the therapeutic effect of four viscosities (50, 150, 430, 670 mPa·s) vs. water plus Omnipaque (<50 mPa·s) and 2 bolus volumes (10 mL and 20 mL) on PAS score at each viscosity level as the primary endpoint to evaluate the therapeutic effect of the thickener, and the kinematics of the oropharyngeal swallow response (OSR) and VFS signs of safety and efficacy of swallow [29] as secondary objectives. Additionally, patient sociodemographic data, anthropometric data, and nutritional status with the Mini Nutritional Assessment (MNA^®^) [30] and the Eating Assessment Tool (EAT-10) [31] were collected, and blood (hemogram, electrolytes, basic liver and kidney profiles, inflammation biomarkers, citrulline) and urine analyses were performed (Figure 1).

Part 2 consisted of two additional weeks where a subset (n = 16) of patients from the first part agreed to follow a daily treatment with TUGE-thickened fluids for 14 days at their residence (home, nursing home or rehabilitation center). Patients were given the study product and instructions to prepare thickened fluids with the dispenser according to their needs based on the VFS results (optimal volume and viscosity) and were asked to register the palatability of the study product, their compliance with the treatment, GI tolerance, and daily fluid intake (mL) with and without the TP in a diary. After the 2-week administration period, the investigator had a 2-day margin to schedule the final visit with the patient. Patients followed the treatment until the last study visit, which included a second blood and urine analysis. Adverse events were also gathered throughout the study (Figure 1).

### 2.4. Study Population

The study population was prospectively recruited from independently living patients with OD referred to the Gastrointestinal Physiology Unit of the Hospital de Mataró (Spain) between May 2021 and May 2022. Recruitment was severely affected by the COVID-19 pandemic. Inclusion criteria were patients over 18 years old, presenting OD according to the V-VST and PAS ≥ 2 in the VFS, patient or caregiver able to record GI symptoms daily, compliance and fluid intake (only for Part 2), and patient able to respond to acceptability questionnaire (only for Part 2). Exclusion criteria were pregnancy, patients with idiosyncratic phenomena or allergic to iodinated contrast media, milk, mustard, egg, or celery, major respiratory disease requiring oxygen or undergoing any type of surgery in the previous three months, current diarrhea, vomiting or abdominal pain, alcohol or drug dependence, COVID-19 disease, unable to comply with the protocol, and obstruction of the gastrointestinal tract (only for Part 2). All participants were informed about the study and signed the informed consent form. The study protocol was approved by the Ethics Committee of the Hospital de Mataró (code 95/20) and was conducted according to the principles and rules laid down in the Declaration of Helsinki and its amendments and following the EU rules for clinical trials on humans (EU Clinical Trial Regulation (EU-CTR, EU No 536/2014)). Finally, adverse events (AEs) and serious adverse events (SAEs) and their relationship with the study product were collected throughout the study according to the WHO and the Uppsala Monitoring Centre (WHO-UMC) category guideline [32]. The relationship of AEs and SAEs to the study product was determined according to the Karch–Lasagna algorithm [33].

### 2.5. Swallowing Evaluation

#### 2.5.1. Volume–Viscosity Swallowing Test (V-VST)

This was used to screen patients with OD for impaired efficacy (impaired labial seal, fractional swallow, oral residue and pharyngeal residue) and/or safety of swallow (signs of cough, wet voice or oxygen desaturation ≥ 3%). The V-VST uses three volumes (5, 10 and 20 mL) and three viscosity levels (<50 mPa·s, 150 mPa·s and 670 mPa·s) in this study and a pulse oximeter to assess clinical signs of impaired safety and efficacy of swallow and OD. This clinical tool, as well as the algorithm and its psychometrics, have been previously and extensively described [28].

#### 2.5.2. Videofluoroscopy (VFS)

**Figure 2:**
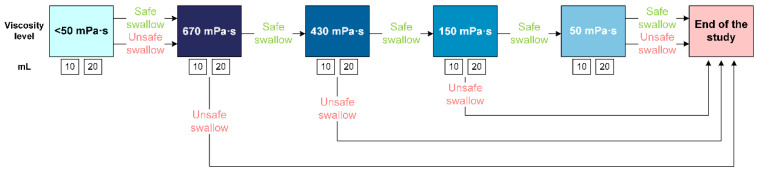
Study intervention algorithm for videofluoroscopy studies in Part 1 according to the results of the rheological studies. mPa·s: millipascal second; mL: milliliters.

This dynamic radiological examination was performed in order to evaluate the severity of OD (signs of both efficacy and safety impairment), the biomechanics of the OSR and the therapeutic effect of ThickenUp^®^ Gel Express. Patients were explored while seated, which included the visualization of the oral cavity, pharynx, larynx, and cervical esophagus. VFS was performed with a XT-20 Toshiba Intensifier (Toshiba Medical Systems Europe, Zoetermeer, The Netherlands) and recorded with a GoPro video camera (GoPro Inc., San Mateo, CA, USA) at 25 frames/s. The study algorithm was designed as an effort test and consisted of a series of first 10 and then 20 mL boluses of the viscosities selected. The protocol began with liquid viscosity < 50 mPa·s and whether or not the swallows performed by the patient were safe, and continued with 670, 430, 150 and 50 mPa·s thickened viscosities (Figure 2). If there was any aspiration (safety rule: PAS ≥ 6) during one of the thickened fluids, the algorithm was stopped and the study concluded for that patient (Figure 2). Signs of impaired safety and efficacy of swallow during VFS were identified. Severity of impaired safety of swallow was scored with the PAS [34]. Timing and biomechanics of the OSR were measured for all the boluses offered to the patients, as previously described, and included the time to laryngeal vestibule closure (LVC), to laryngeal vestibule opening (LVO), to upper esophageal sphincter opening (UESO), kinetic energy (KE) of the bolus, bolus propulsion force, and mean bolus velocity [35].

### 2.6. Hydration Status and Analytical Parameters

#### 2.6.1. Serum and Urine Biochemical Analysis

A blood sample was taken and analyzed for the following parameters: (a) biochemistry parameters and enzymes: glucose, sodium, potassium, calcium, magnesium, chloride, bicarbonate, alanine transaminase (ALT), aspartate transaminase (AST), serum creatinine, C-reactive protein (CRP), citrulline, urea, and urea/creatinine ratio; (b) hematology parameters: hemoglobin, hematocrit, mean red blood cell volume, white blood cell count and platelets. Urine was collected for the determination of urea, urine osmolarity, urine specific gravity and sodium. The reference values used were those defined by Laboratori de Referència de Catalunya [36].

#### 2.6.2. Bioelectrical Impedance Analysis (BIA)

Body composition was measured with InBody S10 (InBody Co., Ltd., Seoul, Republic of Korea). The percentage of total water, extracellular water, intracellular water, cellular mass, fat mass, lean mass, muscle mass and the phase angle (in degrees) were obtained.

### 2.7. Fluid Intake, Compliance and Acceptability

During the 2-week follow-up study treatment, patients were asked to fill in a diary, designed in accordance with the Annex 5 of Guidelines for submitting a nutritional product to the Advisory Committee for Borderline Substances (ACBS) [37], that included:

#### 2.7.1. Fluid Intake and Compliance with the Intervention

The patient or caregiver was instructed to record the total fluid consumption of any type (water, tea, coffee, juice, or others) with and without the TP in a daily diary. Glasses of each fluid type prepared by the patient or the caregiver were registered as well as the final quantity consumed. Each patient was encouraged to take at least 1.5 L of fluid every day. Participants were considered as compliant if they had consumed 78% of the proposed fluid volume recommended in the study protocol.

#### 2.7.2. Acceptability

An acceptability questionnaire was used to rate from 1 (disliked a lot) to 5 (liked a lot) the appearance, smell, taste and texture/mouthfeel of the TP. Additional questions on the use (yes/no) and the difficulty (easy, okay, difficult, impossible) in taking the TP during the follow-up period were also registered.

#### 2.7.3. Gastrointestinal Tolerance

A tolerance diary was also completed by those patients included in Part 2 and revised at the follow-up visit. It included questions on the presence, increase or decrease and duration in the number, size and consistency of stools, vomits, abdominal pain, bloating or distension, retching, burping or flatulence and other symptoms. It also included the registry of changes in patient medication and its relationship with the appearance of the previously described symptoms.

### 2.8. Data Analysis

The age and sex of the patients were taken into account when interpreting the results of the analytical tests. A PAS score of 2 is considered a mild penetration. Even so, PAS 2 penetrations can also be observed in some healthy persons. For this reason: (a) when calculating the prevalence of safe swallows, patients with a PAS ≤ 2 were included; (b) when calculating the prevalence of penetrations, patients with a PAS ≥ 2 were included; (c) a sub-analysis only including patients with more severe impairments in safety of swallow and PAS ≥ 3 as inclusion criteria was also performed. Serum osmolarity was calculated with the following formula [38]:

1.86 × (Na + K) + 1.15 × glucose + urea + 14 (all measures in mmol/L).

### 2.9. Statistical Analysis

All statistical tests were performed with GraphPad Prism 6 (GraphPad Software, San Diego, CA, USA). Continuous variables were compared with a two-way ANOVA test and expressed as mean ± standard deviation (SD). A multiple comparison post-test was performed to describe any differences between groups. Categorical data were compared with the chi-square test and expressed as relative and absolute frequencies. The two-to-two comparisons were made using Fisher’s test. Nonparametric tests were used when appropriate. Significant differences were considered for *p*-value < 0.05.

## 3. Results

### 3.1. Rheological Characterization of TUGE

Four viscosity levels of thickened fluids were assessed by adding 5, 10, 20 or 30 g of TUGE (corresponding to one, two, four or six pushes of the dispenser) to 150 mL of two different fluids, one of water for V-VST and hydration studies and the other including the X-ray contrast for VFS studies. The viscosity at 50 s^−1^ ranged from 56.22 ± 4.23 to 614.20 ± 11.42 mPa·s when TUGE was mixed with 150 mL water for V-VST or daily conditions (Table 1) and from 49.41 ± 2.38 to 672.5 ± 35.62 mPa·s when mixed with 75 g water + 101.2 g Omnipaque for VFS studies (Figure 2 and Table 2).

Oral incubation of TUGE mixed in water in healthy adults caused a non-significant mean reduction in shear viscosity (1.6%) at all thickened levels, with a maximal non-significant reduction of −8.14% from the initial viscosity at the highest viscosity level, showing a strong amylase-resistant behavior for TUGE. Shear rate at 300 s^−1^ caused a significant mean decrease in viscosity (shear thinning) of 72.3%. Both rheological factors affecting viscosity (amylase in the oral phase and shear thinning in the pharyngeal phase) produced a mean decrease in shear viscosity of 72.1%. Regarding the X-ray contrast effect, Omnipaque caused a slight variable effect on the shear viscosity achieved by TUGE with some increase at lower viscosity values and a maximal decrease of 11.25%, and no significant changes in amylase resistance (mean amylase effect: 0.3%) or shear thinning. We did not find any significant difference in α-SA resistance or shear thinning between water vs. X-contrast solutions.

### 3.2. Part 1: VFS Studies

#### 3.2.1. Study Population

**Figure 3:**
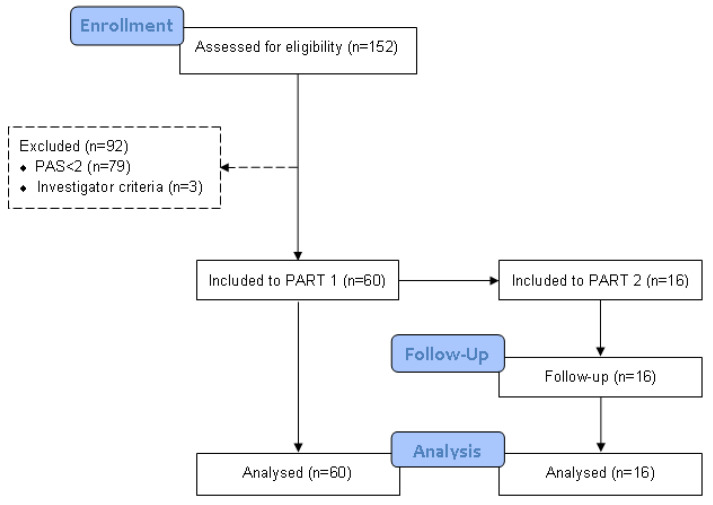
CONSORT flow-chart of the clinical studies. n: sample size; PAS: penetration–aspiration scale.

A total of 152 patients with OD were assessed for eligibility, of which 79 were excluded for presenting PAS < 2 during VFS assessment and three for investigator criteria. Finally, 60 independently living patients with OD and PAS ≥ 2 were included in the study (70.0 ± 11.4 years, 43.3% women) (Figure 3).

#### 3.2.2. Hydration and Nutritional Status

According to MNA^®^, 46.3% of patients were at risk of malnutrition (MNA^®^: 20.6 ± 2.0) while 4.9% were malnourished (MNA^®^: 14.5 ± 0.7). Regarding hydration status, 65.9% of patients with OD included in this study were dehydrated according to urea/creatinine ratio, but this prevalence increased to 75.7% when calculated according to serum osmolarity. Supplementary Table S1 shows the mean results of each serum and urine parameter studied.

#### 3.2.3. Oropharyngeal Dysphagia Status

All patients had OD and presented a positive V-VST for signs of either impaired safety or efficacy of swallow. Prevalence of patients with clinical signs of impaired safety of swallow was 58% and prevalence of patients with signs of impaired efficacy of swallow was 78%; 68% of patients presented both signs. Regarding VFS results, all 60 patients included in the study presented a PAS ≥ 2. All of them (100%) showed signs of efficacy and safety impairment, of which 25% were aspirations and with a mean maximum PAS score of 4.1 ± 2.2. In addition, they showed a moderate delay in airway protection mechanisms and time to LVC (Table 3).

#### 3.2.4. Therapeutic Effect of TUGE on PAS Score (Primary Endpoint)

**Figure 4:**
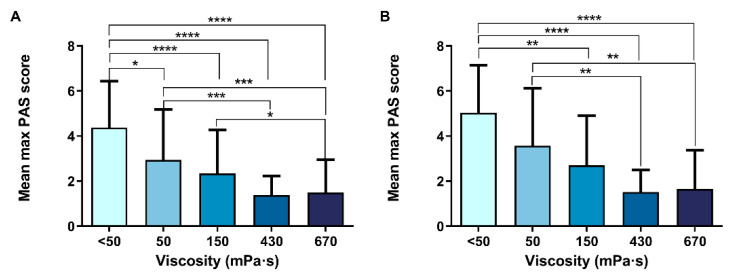
Effect of increasing shear viscosity on safety of swallow assessed by PAS (mean maximum PAS score) in those patients with PAS ≥ 2 (**A**) and with PAS ≥ 3 (**B**). PAS: penetration–aspiration scale; mPa·s: millipascal per second; *: *p* < 0.05; **: *p* < 0.01; ***: *p* < 0.001; ****: *p* < 0.0001.

Increasing fluid viscosity with TUGE significantly decreased the mean maximum PAS score and increased the prevalence of patients with PAS 1 or 2 (decreasing the severity of safety impairments as a consequence) compared with liquid in a shear viscosity-dependent manner in those with PAS ≥ 2 (Figure 4A) and also in those with PAS ≥ 3 (Figure 4B). The therapeutic range in those with PAS ≥ 3 was 150–670 mPa·s.

#### 3.2.5. Therapeutic Effect of TUGE on VFS Signs of Impaired Safety

**Figure 5:**
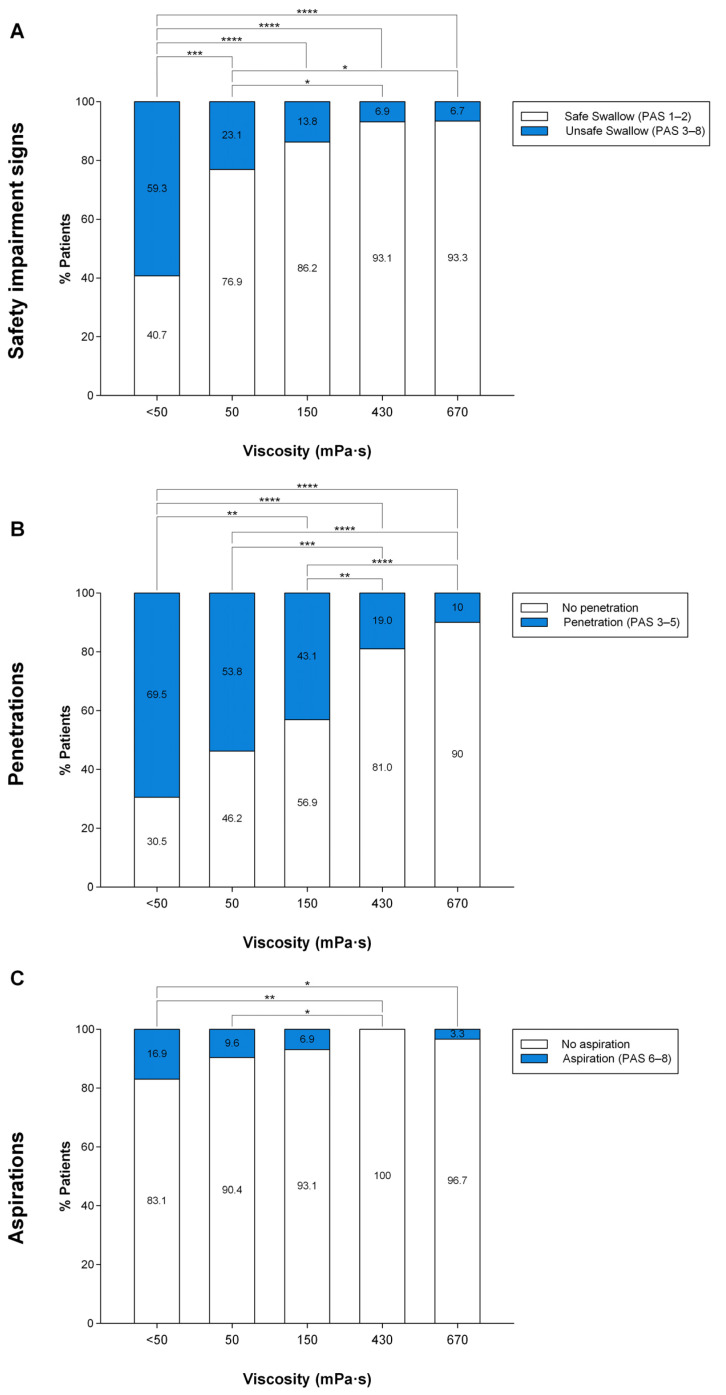
Graphical representation of the therapeutic effect of increasing bolus viscosity on the prevalence of patients with safe/unsafe swallows (**A**), penetrations (**B**) and aspirations (**C**) at each viscosity level in patients with PAS ≥ 2. PAS: penetration–aspiration scale; mPa·s: millipascal per second; *: *p* > 0.05; **: *p* < 0.01; ***: *p* < 0.001; ****: *p* < 0.0001.

**Figure 6:**
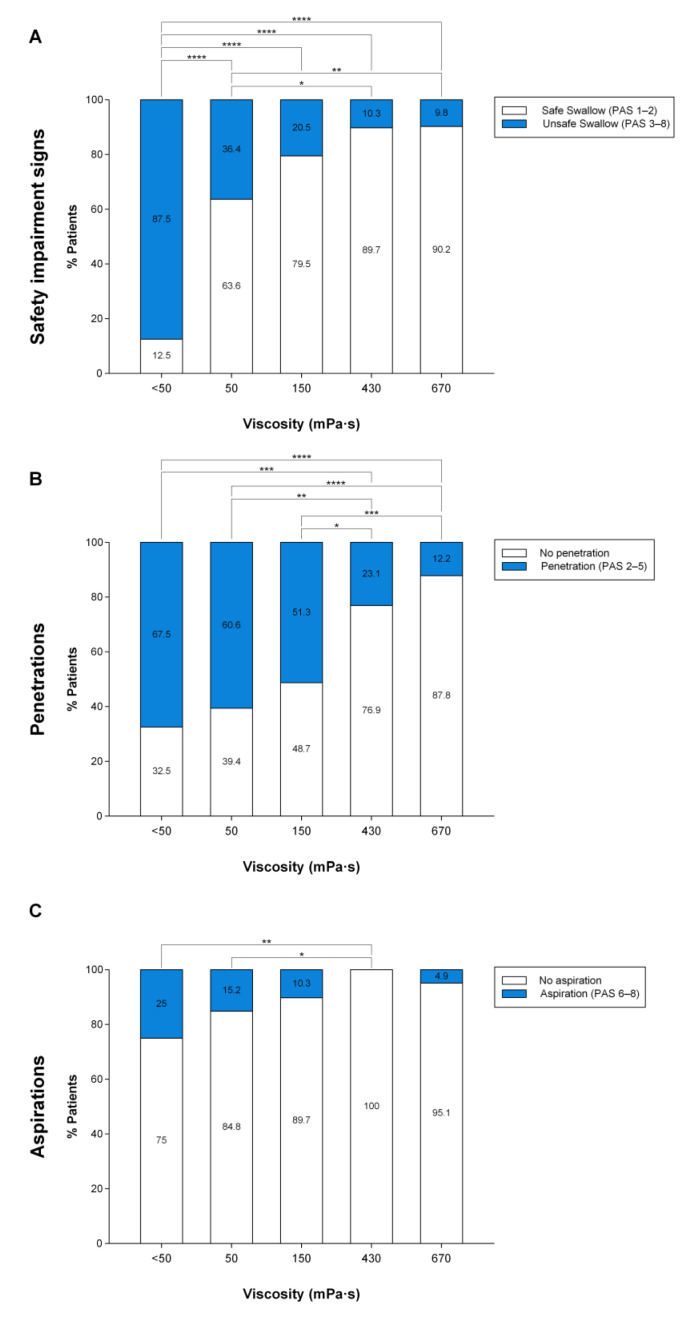
Graphical representation of the therapeutic effect of increasing bolus viscosity on the prevalence of patients with safe/unsafe swallows (**A**), penetrations (**B**) and aspirations (**C**) at each viscosity level in patients with PAS ≥ 3. PAS: penetration–aspiration scale; mPa·s: millipascal per second; *: *p* > 0.05; **: *p* < 0.01; ***: *p* < 0.001; ****: *p* < 0.0001.

At < 50 mPa·s, 59.3% patients with OD and PAS ≥ 2 as inclusion criteria presented impaired safety of swallow, 69.5% of whom presented penetrations and 16.9%, aspirations. In contrast, in patients with more severe OD and inclusion criteria PAS ≥ 3, 87.5% presented impaired safety of swallow, 67.5% of whom presented penetrations and 25% aspiration. Fluid thickening with TUGE caused a significant shear viscosity-dependent increase in the prevalence of patients with safe swallows (Figure 5A and Figure 6A) and greatly reduced the prevalence of both penetrations (Figure 5B and Figure 6B) and aspirations (Figure 5C and Figure 6C), in patients with OD and PAS ≥ 2 as inclusion criteria (n = 60) and also in patients with more severe OD and PAS ≥ 3 (n = 41) as inclusion criteria.

**Figure 7:**
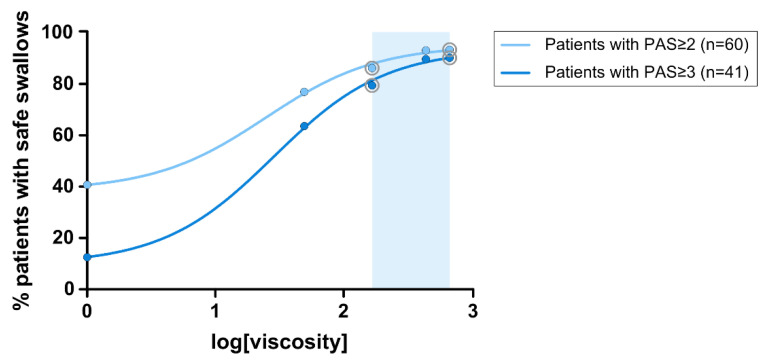
Dose–response curve of the viscosity levels on the prevalence of patients with safe swallows in patients with PAS ≥ 2 and PAS ≥ 3. Blue band indicates therapeutic range (150–670 mPa·s) and gray circles the optimal viscosity values.

When patients with OD and PAS ≥ 2 as inclusion criteria were analyzed, we found 63.3% of patients needed to drink thickened fluids to swallow safely, of whom up to 50% needed 150 mPa·s and 13.3% needed 670 mPa·s. In patients with more severe OD and PAS ≥ 3 as inclusion criteria, 92.7% needed to drink thickened fluids, of whom up to 73.2% needed 150 mPa·s and 19.5% needed 670 mPa·s to swallow safely. Taking into account these results, the therapeutic range of TUGE goes from 150 to 670 mPa·s, 150 and 670 mPa·s being the two optimal viscosity values to provide a safe swallow to all patients with mild and severe OD with only two viscosity levels (Figure 7).

#### 3.2.6. Therapeutic Effect of TUGE on VFS Signs of Impaired Efficacy

At the higher viscosity level (670 mPa·s), the prevalence of patients with oral residue was 52.5% and all of these patients had a coating residue, while the prevalence of patients with pharyngeal residue was 64.4% (37.3% coating, 27.1% pooling). Overall, VFS results showed a similar prevalence of patients with oral or pharyngeal residue. However, pharyngeal residue was significantly more severe (pooling) than oral residue (coating, *p* = 0.001).

**Figure 8:**
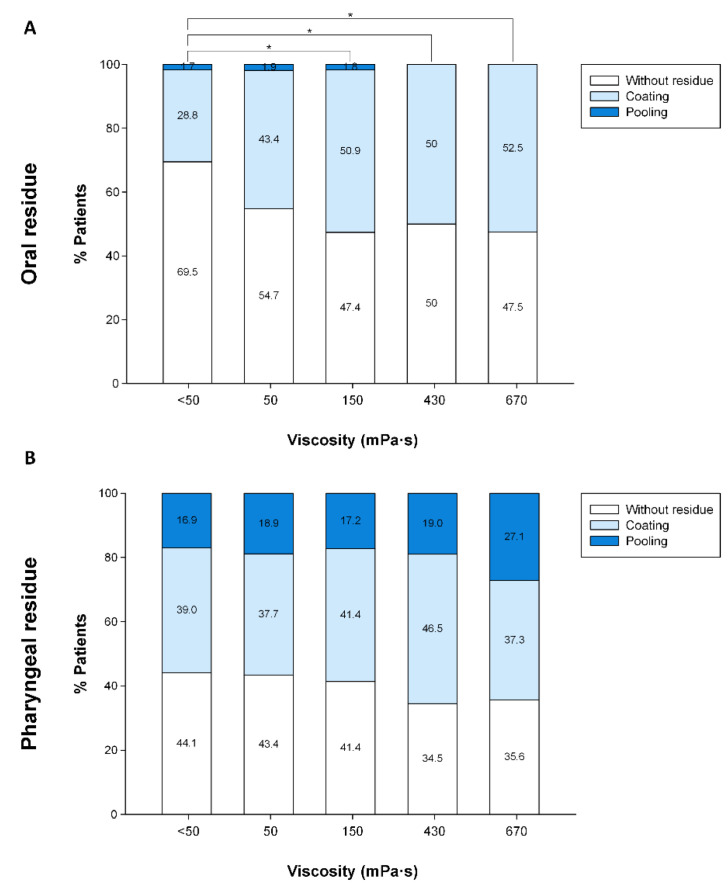
Graphical representation of the effect of increasing viscosity on the prevalence of oral (**A**) and pharyngeal (**B**) residue. mPa·s: millipascal per second; *: *p* < 0.05.

**Figure 9:**
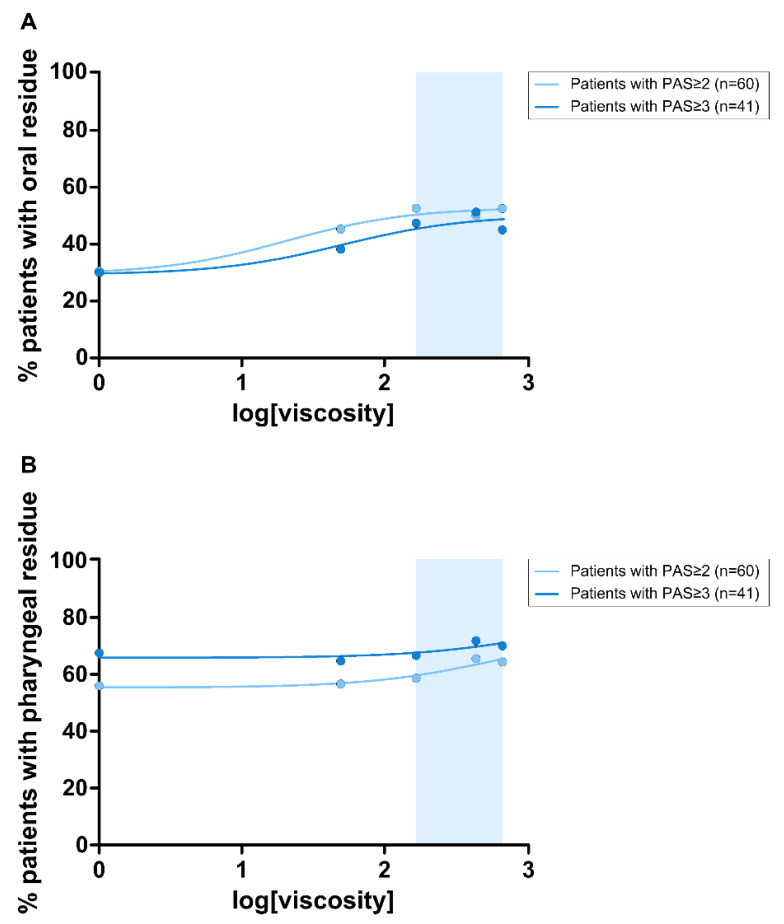
Dose–response curve of the viscosity levels on the prevalence of patients with oral (**A**) and pharyngeal (**B**) residue in patients with PAS ≥ 2 and patients with PAS ≥ 3. Blue band indicates therapeutic range (150–670 mPa·s).

Fluid thickening with TUGE at higher viscosity levels (150, 430 and 670 mPa·s) significantly increased the prevalence of patients with oral residue compared to liquid (Figure 8A) without viscosity-dependent effects (Figure 9A). In contrast, no significant effect of increasing viscosity was observed on prevalence of patients with pharyngeal residue (Figure 8B and Figure 9B).

#### 3.2.7. Effect of Fluid Thickened with TUGE on Timing and Kinematics of OSR

**Figure 10:**
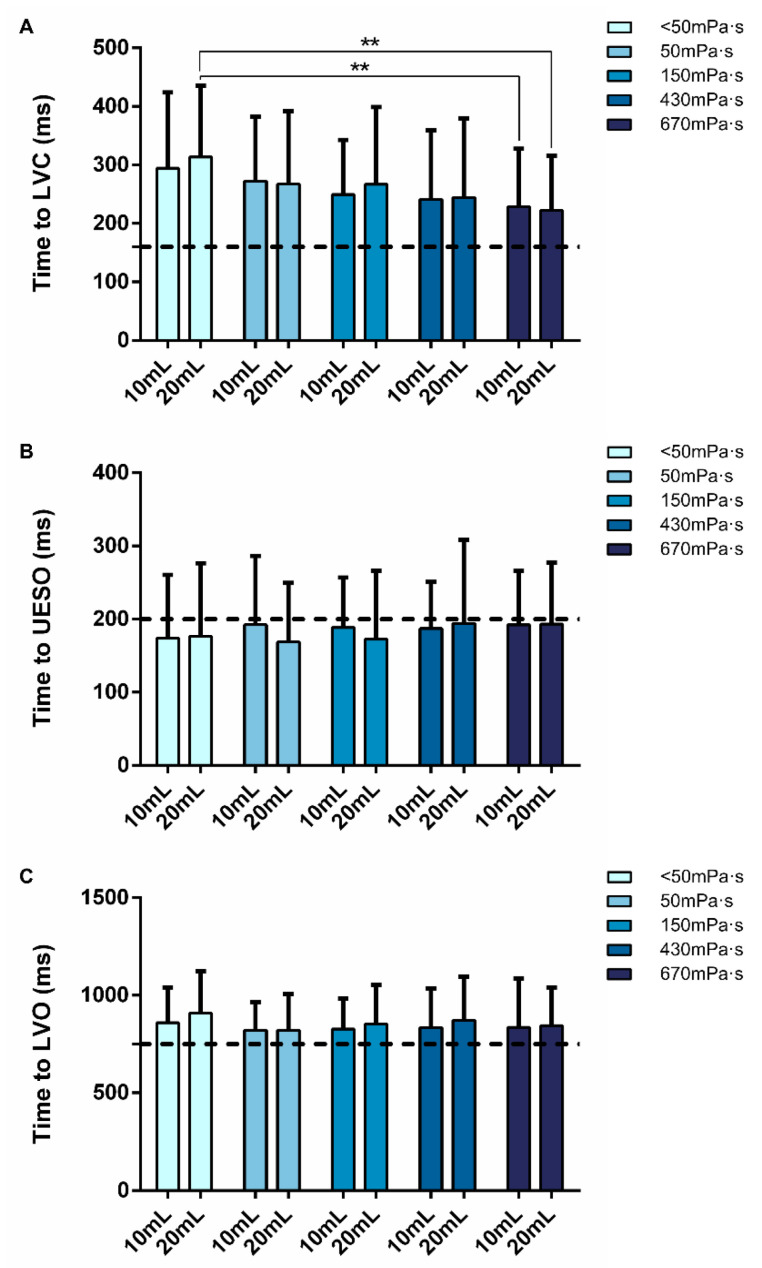
Graphical representation of the effect of bolus volume and viscosity on the timing of OSR: Time to laryngeal vestibule closure (LVC) (**A**), to upper esophageal sphincter opening (UESO) (**B**) and to laryngeal vestibule opening (LVO) (**C**). Broken lines mark the mean time described in healthy volunteers [18,30]. mL: milliliters; ms: milliseconds; **: *p* < 0.01.

Patients included in this study presented impaired timing of OSR with delayed time to LVC (294.5 ± 129.4 ms) and prolonged swallow response (glosso palatal junction–LVO: 859.9 ± 180.4 ms). In contrast, no alterations in time to UESO (174.0 ± 86.5 ms) were observed. Increasing bolus viscosity from < 50 mPa·s to 50, 150 and 430 mPa·s did not affect time to LVC. In contrast, at the highest viscosity levels (670 mPa·s), a significant reduction in time to LVC (*p* = 0.003) was observed. TUGE did not affect time to UESO or to LVO at any viscosity level tested. No effect was observed with increasing volume at the same viscosity level (Figure 10).

**Figure 11:**
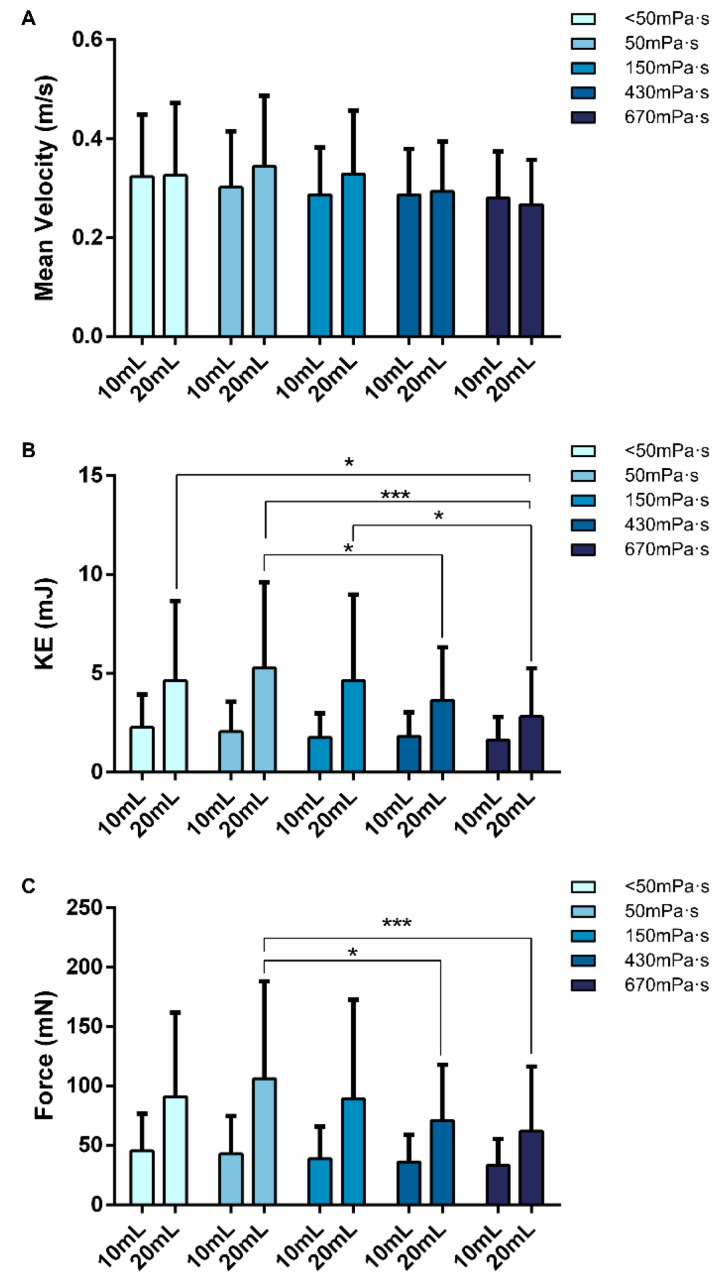
Graphical representation of the effect of increasing volume and viscosity on mean bolus velocity (**A**), kinetic energy (KE) (**B**) and propulsion force (**C**). mL: milliliters; m/s: meters per seconds; mJ: millijoule; mN: millinewton; *: *p* < 0.05; ***: *p* < 0.001.

Regarding the kinematics of OSR, the two highest viscosity levels (430 and 670 mPa·s) led to a significant reduction in bolus kinetic energy and bolus propulsion force. However, no significant effect of increasing viscosity on mean pharyngeal bolus velocity was observed, nor was an effect of increasing bolus volume found for any of the kinematic parameters assessed (Figure 11).

### 3.3. Part 2: Effect of TUGE on Hydration Status, Acceptability and GI Tolerance

#### 3.3.1. Study Population

The first 16 patients (74.4 ± 10.2 years, 37.5% women) from Part 1 who were willing to take the study product for two weeks and who were able (or their caregiver) to complete the daily diary and the ACBS questionnaires (palatability and GI tolerance) were included in Part 2. These patients were at risk of malnutrition according to MNA (22.3 ± 3.7), and most of them were dehydrated (81.3% according to urea/creatinine ratio, 86.7% according to serum osmolarity). Regarding their OD status, all patients had impaired efficacy (81.25% oral residue, 68.75% pharyngeal residue) and safety of swallowing (43.8% aspirations) and a mean PAS score of 5.5 ± 2.2 in VFS study. They also showed delayed time to LVC (303.5 ± 110.7 ms) and to LVO (863.1 ± 82.9 ms).

#### 3.3.2. Compliance and Therapeutic Effect on Hydration Status

According to the individual VFS results, the largest group of patients in this subgroup were prescribed to drink thickened fluids at 150 mPa·s (44%), followed by 430 mPa·s (37%) and 50 mPa·s (19%). After the 14-day intervention, a very high adherence (93–100%) was observed and the mean amount drunk was 1488 mL/day, of which 87.3% was thickened fluids with TUGE. This resulted in an improvement of several biochemical parameters (hemoglobin, hematocrit, urea, calcium and magnesium). No significant changes in sodium serum concentration were observed, and only a slight non-significant increase in sodium urine concentration was observed (Table 4). The prevalence of dehydrated patients was also reduced to 43.8% and 73.3% according to the urea/creatinine ratio and serum osmolarity, respectively. No significant effects on urine nor BIA parameters were observed.

#### 3.3.3. Acceptability and Gastrointestinal Tolerance

For the acceptability test, patients rated the appearance (56%), smell (44%) and taste (38%) as “neutral”, while texture was rated as “like” by 44% (Table 5). In addition, 62% said that TUGE-thickened fluids were easy to drink under normal conditions, while the other 38% said that it was “Okay”.

**Figure 12:**
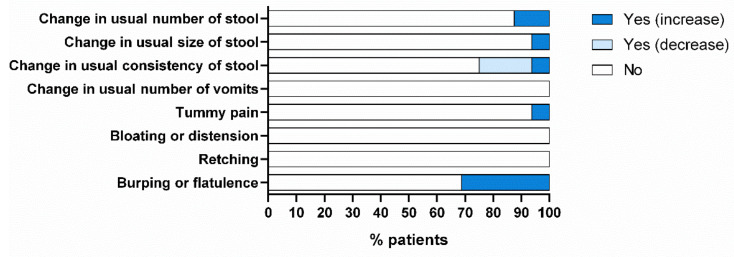
Results from the gastrointestinal tolerance test.

Regarding the GI tolerance test, patients reported no serious side effects. The most frequently reported adverse effects were change in usual consistency of stool (19% softer and 6% harder) and increase in burping or flatulence (33%). Change in usual consistency of stool has been classified as “unlikely related to the study product”. In contrast, the increase in burping or flatulence has been classified as “probably related to the study product”. The other side effects reported by the patients (change in usual number (13%) and size (6%) of stool and tummy pain (6%)) could not be related to the product under study (Figure 12).

## 4. Discussion

The main results of the present study show that TUGE, a xanthan- and acacia-gum-based, concentrated, liquid, thickening product unaffected by α-SA, has a therapeutic effect that strongly improves safety of swallow in a viscosity-dependent manner at a therapeutic range of 150–670 mPa·s, without affecting pharyngeal residue, in independently living older patients with mild OD. The mechanism of action of TUGE was mainly compensatory without major effects on airway protection mechanisms at 150–430 mPa·s and adding improved time to LVC only at high viscosity levels (670 mPa·s). We also found 14-day treatment with TUGE to be safe and well tolerated, and improved hydration status in this phenotype of patients with OD. In addition, its liquid form was convenient to prepare, as the TP mixes easily into beverages, simplifying dissolution. Taken together, we believe fluid thickening with TUGE allows safe and well-tolerated thickened fluid (TF) therapy and optimal fluid volume intake to improve the hydration status of independently living older patients with OD [10].

Few studies have reported the prevalence of dysphagia in independently living older people. In an earlier study, we used V-VST in a population-based, cross-sectional study to assess the true prevalence of OD, impaired efficacy of swallowing, impaired safety of swallowing, and aspiration in the independently living older population (aged 70 and older) from a primary care center database in Mataró (Barcelona, Spain) [39]. The following true population prevalences were estimated: OD, 23.0% (33.0% in ≥ 80 vs. 16.6% in 70–79). We also explored the impact of OD on nutritional and respiratory complications of these citizens in a 1-year follow-up prospective study and found that impaired efficacy of swallow was a risk factor for malnutrition and nutritional risk, and OD as a whole was a risk factor for loss of functional capacity in independently living persons 70 years old or over. Otherwise, the annual incidence of low respiratory tract infections (LRTI) was higher in persons with basal signs of impaired safety of swallow in comparison with those without such signs. These results show that a large number of older people in the community are at risk of nutritional and respiratory complications due to swallowing disorders that are under-diagnosed and under-treated [39,40]. We have also recently found that dehydration was a highly prevalent complication in older patients with chronic OD [10], and although there is scientific evidence on the positive effect of TF therapy on the hydration status of patients with OD, strict monitoring of fluid volume intake is essential due to the low consumption of TF in these patients [10]. In our present study, up to 75.7% of our patients were dehydrated according to serum osmolarity. Taken together, all these results show that the independently living older population with OD is prevalent, at risk of dehydration and severe respiratory complications and a clear target for TF treatment to improve their hydration status.

In the present study, V-VST was also used to screen independently living patients with OD (with signs of impaired safety and/or efficacy of swallowing) and VFS to assess those with signs of impaired safety of swallow to be included in the therapeutic VFS study (Part 1). Up to 60% of the patients included in the study showed signs of impaired safety during VFS liquid series, 25% being aspirations. These patients had a significant delay in the time to LVC (294.5 ± 129.4 ms) and to LVO (859.9 ± 180.4 ms) when compared to healthy adults (LVC < 160 ms; LVO < 750 ms in HS). In addition, this cohort of patients showed reduced bolus propulsion force and low bolus KE. Regarding signs of impaired efficacy, 71.7% and 76.3% presented oral and pharyngeal residue, respectively. All these data show that the population of independently living older patients included in the study presents mild to moderate dysphagia in comparison to hospitalized frail older patients with OD. For this reason, we performed a subanalysis of patients with OD and impaired safety of swallow PAS ≥ 3, selecting patients with more severe OD [18,27].

TUGE is a gum-based thickening product that was designed to be easy to use (convenient preparation, simple and consistent dosing in all beverages), fast (reaching the needed viscosity quickly), accurate, with a dosing system that saves measuring manually, and stable over time. In the present study, four viscosities were used apart from liquid. The mean viscosity (at 50 s^−1^ and 25 °C and using a protocol that has been validated internationally [41]) of each level was 56.22 ± 4.23, 154.20 ± 0, 407.23 ± 11.66 and 614.20 ± 11.42 mPa·s when gel from one, two, four or six pushes on the TUGE dispenser, respectively, were mixed in 150 mL of water (hydration conditions and V-VST). For the other mixture, we obtained 49.41 ± 2.38, 154.83 ± 10.22, 439.33 ± 11.72 and 672.5 ± 35.62 mPa·s when gel from one, two, four or six pushes on the TUGE dispenser was mixed in 75 g of water and 101.2 g of Omnipaque for VFS studies. We described each level tested in this VFS clinical trial using SI units in mPa·s as follows: < 50, 50, 150, 430 and 670 mPa·s. Our results further confirm the need to use SI units to label the viscosity levels of thickening agents [23], essential in clinical trials assessing their therapeutic effect.

Our in vitro rheologic results for TUGE are consistent with the behavior of a gum-based thickening product resistant to salivary alpha-amylase (mean viscosity reduction of 0.3%), with a reduction in viscosity of 72.3% by a shear rate at 300 s^−1^ (shear thinning effect during pharyngeal bolus flow) and with thickening properties unaffected by Omnipaque X-ray contrast used in VFS studies. We have studied the shear rate spectrum for the whole swallowing process in humans, estimated to range between 1 and 1000 s^−1^ [41], and the two major landmarks proposed as replicating the swallowing process in patients with OD. The first landmark is in the oral cavity, and 50 s^−1^ has been recommended [42,43]; the second landmark is in the pharynx (specifically in the mesopharynx) where the head of the bolus reaches the laryngeal vestibule and can be aspirated. At this specific anatomic point, shear rate has been determined at 300 s^−1^ [41].

The main aim of the present study was to assess the therapeutic effect of ThickenUp^®^ Gel Express on PAS score, safety and efficacy of swallow, and the kinematics of swallow response (assessed by VFS) and on hydration status (serum and urine analysis, BIA and fluid intake). Regarding the therapeutic effect on swallowing safety, the study clearly shows that increasing fluid viscosity significantly reduced the mean maximum PAS score at 150–670 mPa·s; therefore, the threshold effect on safety of swallow was observed at 150 mPa·s. Prevalence of patients with safe swallows was significantly increased in a shear viscosity-dependent manner, moving from 40.7% with liquid to 93.3% at 670 mPa·s when the inclusion criteria was PAS ≥ 2, and from 12.5% to 90.2% when the inclusion criteria was PAS ≥ 3. In patients with PAS ≥ 2, the prevalence of penetrations (69.5%) was significantly reduced at 150 (43.1%), 430 (19%) and 670 (10%) mPa·s, while the prevalence of aspirations (16.9%) was significantly reduced only at 430 mPa·s (0%) and 670 mPa·s (3.3%). In contrast, in patients with PAS ≥ 3, only the viscosity at 430 mPa·s reduced the prevalence of both penetrations (23.1%) and aspirations (0%) when compared to liquid (penetrations: 67.5%, aspirations: 25%). At 670 mPa·s, the prevalence of penetrations was significantly reduced to 12.2%. With these data, we determined that the therapeutic viscosity range of TUGE ranges from 150 to 670 mPa·s, with an improvement in airway protection mechanisms by reducing time to LVC at 670 mPa·s. These are the two optimum viscosities needed to provide a safe swallow to almost all independently living patients: 150 (with the threshold effect which guarantees high treatment compliance) and 670 mPa·s, which provides maximal safety and improved physiology to these patients.

Regarding signs of impaired efficacy, oral residue caused by TUGE was mild, “coating” and worsened with increasing viscosity when compared to liquid, while pharyngeal residue was more severe “pooling”. This small effect of TUGE on the increase in the prevalence of oropharyngeal residue is not shear-viscosity-dependent, and these results are also consistent with other gum-based thickeners [18,24]. Our study also demonstrates that the mechanism of action of TUGE on safety of swallow is mainly compensatory, as time to LVC remained unchanged until using the highest 670 mPa·s bolus viscosity (that caused a 27% reduction in time to LVC). This viscosity level of 670 mPa·s also caused a reduction in bolus propulsion force and bolus kinetic energy without changes in mean bolus velocity. In addition, no effects on timing of digestive configuration (time to UESO) nor overall duration of the swallow response (LVO) were observed. Following our results, we suggest that the mechanisms of action of TUGE on safety of swallow is dual: (a) mainly compensatory and depending on the intrinsic rheological characteristics of this thickening agent and not associated with any major change in oropharyngeal physiology at mid viscosity levels, and (b) caused an improvement in airway protection mechanisms and the addition of changes in swallow physiology at 670 mPa·s. A future study on the extensional rheological properties of the compound will help understand the full picture of its mechanisms of action.

Finally, those patients that were included in Part 2 showed several improvements in the hydration-related serum analytical parameters. After a 14-day intervention, we observed a significant reduction in the hematocrit and hemoglobin and also of the concentration of calcium, magnesium and urea. However, it is important to note that the prevalence of dehydration varies according to the parameter used to calculate it, going from 81.3% to 43.8% if it was calculated with the urea/creatinine ratio or from 86.7% to 73.3% if calculated with the serum osmolarity, as specified in the ESPEN guidelines [38]. All these improvements were probably due to the high adherence (93–100%) and compliance of patients with the recommended fluid intake, reaching a mean fluid intake of 1488 mL/day, of which 87.3% were TUGE-thickened fluids. In addition, no differences were observed (for fluid intake or for analytical parameters) for the different viscosity levels prescribed to the patients. This suggests that the high prevalence of dehydration in patients with OD is caused by the swallow impairment itself and related to its severity, and not related to the use of thickening products; it further confirms that appropriate volumes of thickened fluids can significantly improve hydration status of older patients [10]. We believe that the essential point to improve hydration status of older patients with dysphagia is to use this new generation of gum-based thickening agents like TUGE and to monitor and encourage the appropriate volume and viscosity of safe fluid intake, instead of arguing against the use of thickening agents [44].

Regarding tolerance and adverse events, most of the patients reported that it was easy to drink thickened fluids with TUGE and rated the smell, taste and texture positively. Regarding GI tolerance, the only side effect that was probably related to the study product was the increase in burping or flatulence (reported by 33% of patients), as the introduction of TUGE was the only change in their dietary habits during the 14-day intervention. On the other hand, change in usual consistency of stool has been classified as “unlikely related to the study product” as it was reported within 48 h after VFS assessment, and this is a common side effect linked to the administration of the X-ray contrast, and the change in usual number (13%) and size (6%) of stool and abdominal pain (6%)) could not be related to the product under study due to lack of data [33]. Finally, for a daily intake of 1500 mL per day, ThickenUp^®^ Gel Express will provide 500 mg of sodium per day at 150 mPa·s and 1500 mg at 670 mPa·s, below the Reference Nutrients Intake of sodium in UK and the recommendations of the European Society of Cardiology. To assess the effect on the biochemical parameters, sodium has been assessed before and after 14-day intervention with TUGE in the ACBS sub-study of clinical study NCT04741581. The sub-study clearly showed that the values of sodium did not change during the intervention after drinking an average fluid intake of 1488 mL/day with TUGE. However, we recommend that care should be taken to treat the more frail and polymorbid older patients with severe OD at high viscosity levels for long periods of time, especially those with cardiovascular events or renal insufficiency who will need a specific and individualized risk-benefit analysis.

The study has some limitations. First, the recruitment of patients was severely affected by the COVID-19 pandemic. This fact caused the recruitment to be lower than expected. In addition, it would also be interesting to evaluate the hydration status with a medium and long-term intervention. Future long-term multicentric studies will help assess the clinical impact of fluid thickening with TUGE in the improvement of the clinical outcomes of patients with OD in terms of respiratory complications, readmissions and mortality.

## 5. Conclusions

ThickenUP^®^ Gel Express is a xanthan- and acacia-gum-based concentrated liquid thickener unaffected by α-SA and affected by up to 70% by pharyngeal shear thinning. Its therapeutic range in independently living older patients with mild OD goes from 150 to 670 mPa·s. The optimal values to provide a safe swallow to most patients of this phenotype, using only two viscosity levels and the V-VST to prescribe them, are 150 and 670 mPa·s. The therapeutic effect of TUGE is mainly through compensatory mechanisms, significantly reducing the PAS score and increasing the prevalence of patients with a safe swallow in a shear-viscosity-dependent-manner at 150 to 430 mPa·s. At the highest viscosity level assessed in the present study (670 mPa·s), we also observed an improvement in airway protection mechanisms. Finally, 14-day intervention with ThickenUP^®^ Gel Express thickened fluids was well tolerated and improved the hydration status of patients with OD without inducing serious GI adverse events.

## Figures and Tables

**Table 1:** Viscosity, shear rate effect and alpha-amylase effect for each viscosity level achieved by TUGE in water for V-VST and hydration studies.

g TUGE /150 mL Water	Viscosity (mPa·s) at 50 s^−1^	Viscosity (mPa·s) at 50 s^−1^ after Oral Incubation	α-Amylase Effect (%)	Viscosity (mPa·s) at 300 s^−1^	Shear Thinning Effect (%)
5	56.22 ± 4.23	56.10 ± 5.09	−0.29	16.22 ± 0.88	−71.15
10	154.20 ± 0	155.12 ± 18.81	0.59	37.69 ± 2.03	−75.56
20	407.23 ± 11.66	374.10 ± 34.20	−8.14	94.61 ± 0.87	−76.77
30	614.20 ± 11.42	624.03 ± 36.43	1.60	143.8 ± 0.57	−76.60

TUGE: ThickenUp^®^ Gel Express; g: grams; mPa·s: millipascal per second; mL: milliliters; s^−1^: reciprocal seconds.

**Table 2:** Viscosity, shear rate effect and alpha-amylase effect for each viscosity level achieved by TUGE in water + Omnipaque X-ray Contrast for VFS studies.

g TUGE/75 g Water + 101.2 g Omnipaque	Viscosity (mPa·s) at 50 s^−1^	Viscosity (mPa·s) at 50 s^−1^ after Oral Incubation	α-Amylase Effect (%)	Viscosity (mPa·s) at 300 s^−1^	Shear Thinning Effect (%)
5	49.41 ± 2.38	58.62 ± 3.48	18.64	18.74 ± 0.11	−71.89
10	154.83 ± 10.22	152.29 ± 5.11	−1.64	43.53 ± 3.95	−71.89
20	439.33 ± 11.72	408.81 ± 9.29	−6.95	131.30 ± 31.27	−70.11
30	672.5 ± 35.62	596.86 ± 17.94	−11.25	166.2 ± 1.70	−75.29

TUGE: ThickenUp^®^ Gel Express; g: grams; mPa·s: millipascal per second; s^−1^: reciprocal seconds.

**Table 3:** VFS signs of safety and efficacy impairment and timing of oropharyngeal swallow response of all the patients included in the clinical studies. Results are expressed as mean ± standard deviation unless specifically indicated.

**Impaired efficacy (%)**	**100**
Impaired labial seal	5.0
Piecemeal deglutition	88.3
Oral residue	71.7
Pharyngeal residue	76.3
**Impaired safety (%)**	100
Penetrations PAS 2–5 (%)	96.7
Aspirations PAS 6–8 (%)	25.0
**Mean max. PAS score**	4.1 ± 2.2
**Time to LVC (ms)**	294.5 ± 129.4
**Time to UESO (ms)**	174.0 ± 86.5
**Time to LVO (ms)**	859.9 ± 180.4

PAS: penetration-aspiration scale; LVC: laryngeal vestibule closure; UESO: upper esophageal sphincter opening; LVO: laryngeal vestibule opening; ms: milliseconds.

**Table 4:** Pre- and post-intervention serum hematology and biochemistry, urinalysis and BIA results of the patients included in Part 2 of the study. Results are expressed as mean ± SD, except when specifically stated.

	Pre-Intervention	Post-Intervention	*p*-Value
**Serum: hematology**			
Hemoglobin (g/dL)	14.3 ± 1.5	13.9 ± 1.6	0.016
Hematocrit (%)	42.8 ± 4.2	41.8 ± 4.3	0.040
Mean red blood cell volume (fL)	91.0 ± 4.8	86.3 ± 22.0	0.623
White blood cell count (u/µL)	6.7 ± 1.7	6.6 ± 1.9	0.831
Platelets (u/µL)	224.1 ± 53.6	216.6 ± 52.1	0.374
**Serum: biochemistry**			
Sodium (mmol/L)	141.0 ± 2.9	140.9 ± 3.3	0.899
Potassium (mmol/L)	4.4 ± 0.4	4.3 ± 0.4	0.283
Calcium (mg/dL)	9.7 ± 0.6	9.4 ± 0.3	0.028
Magnesium (mg/dL)	2.1 ± 0.2	2.0 ± 0.2	0.006
Chloride (mmol/L)	102.0 ± 3.2	102.4 ± 4.0	0.387
Bicarbonate (mmol/L)	27.1 ± 3.3	27.4 ± 3.8	0.264
ALT (U/L)	21.5 ± 17.3	22.2 ± 20.7	0.969
AST (U/L)	21.8 ± 13.2	20.9 ± 9.0	0.989
Creatinine (mg/dL)	0.9 ± 0.3	0.9 ± 0.2	0.591
CRP (mg/dL)	0.7 ± 1.1	0.3 ± 0.3	0.208
Citrulline (µmol/L)	43.8 ± 21.9	44.7 ± 13.9	0.867
Urea (mg/dL)	40.6 ± 13.1	36.9 ± 11.3	0.039
Urea/creatinine ratio (mg/dL)	46.1 ± 12.1	43.2 ± 13.2	0.144
% Dehydrated *	81.3	43.8	0.066
Osmolarity (mmol/L)	298.8 ± 5.3	298.4 ± 6.7	0.599
% Dehydrated **	86.7	73.3	0.651
**Urine: Urinalysis**			
Urine osmolarity (mOsm/Kg)	530.1 ± 215.4	501.9 ± 188.4	0.401
Urine specific gravity (g/mL)	1.0 ± 0.0	1.0 ± 0.0	0.215
Sodium (mmol/L)	95.0 ± 50.9	106.8 ± 42.8	0.348
**BIA**			
Free fat mass (%)	49.0 ± 7.3	49.2 ± 7.7	0.813
Body fat (%)	29.0 ± 6.4	27.6 ± 8.0	0.563
Body cell mass (%)	26.5 ± 4.7	31.6 ± 4.9	>0.999
TBW (%)	36.3 ± 5.5	36.5 ± 5.7	0.844
ECW (%)	14.3 ± 2.3	14.4 ± 2.3	0.781
ICW (%)	21.9 ± 3.3	22.1 ± 3.4	0.844

g: grams; dL: deciliter; fL: femtoliter; U: units; µL: microliter; mmol: millimol; L: liter; mg: milligrams; µmol: micromole; mOsm: milliosmoles; Kg: kilogram; g: grams; mL: milliliter; ALT: Alanine Transaminase; AST: Aspartate Transaminase; CRP: C-reactive protein; BIA: Bioimpedance; TBW: total body water; ECW: extracellular water; ICW: intracellular water. * urine/creatinine ratio > 45 mg/dL and ** a serum osmolarity > 295 mmol/L were used to define dehydration.

**Table 5:** Answers to the acceptability questionnaire of the patients included in Part 2 (*n* = 16).

	Texture	Taste	Smell	Appearance
Like a lot 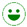	12%	31%	37%	13%
Liked 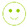	44%	25%	19%	25%
In between 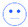	25%	38%	44%	56%
Dislike 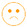	19%	6%	0%	6%
Dislike a lot 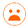	0%	0%	0%	0%

## Data Availability

The data are not publicly available due to privacy and ethical restrictions.

## Supplementary Materials

The following supporting information can be downloaded at: https://www.mdpi.com/article/10.3390/nu15214621/s1, Table S1: Results of each serum and urine parameter studied. Results are expressed as mean ± standard deviation unless specifically indicated.
