# Supplementary material for: Therapeutic Effect on Swallowing Function and on Hydration Status of a New Liquid Gum-Based Thickener in Independently-Living Older Patients with Oropharyngeal Dysphagia
Source: Nutrients. 2023 Oct 31;15(21):4621. doi: 10.3390/nu15214621 (PMC10647578; doi:10.3390/nu15214621)
Supplement: Supplementary file 1 [file nutrients-15-04621-s001.zip › nutrients-2649405-supplementary.pdf]

Supplementary table S1. Results of each serum and urine parameter studied. Results are expressed as mean ± standard deviation unless specifically indicated.

**Serum: hematology**	
Hemoglobin (g/dL)	14.3 ± 1.4
Hematocrit (%)	42.6 ± 3.9
Mean red blood cell volume (fL)	91.1 ± 5.2
White blood cell count (u/μL)	7.7 ± 3.4
Platelets (u/μL)	234.8 ± 65.1
**Serum: biochemistry**	
Sodium (mmol/L)	140.4 ± 3.2
Potassium (mmol/L)	4.4 ± 0.4
Calcium (mg/dL)	9.8 ± 0.4
Magnesium (mg/dL)	2.1 ± 0.2
Chloride (mmol/L)	101.5 ± 3.6
Bicarbonate (mmol/L)	27.4 ± 3.1
ALT (U/L)	18.7 ± 12.4
AST (U/L)	20.1 ± 9.6
Creatinine (mg/dL)	0.9 ± 0.3
CRP (mg/dL)	0.6 ± 1.1
Citrulline (μmol/L)	41.2 ± 18.0
Urea (mg/dL)	38.7 ± 12.8
Urea/creatinine ratio (mg/dL)	44.9 ± 10.6
% Dehydrated *	68.3
Osmolarity (mmol/L)	297.5 ± 7.2
% Dehydrated **	73.6
**Urine: Urinalysis**	
Urine osmolarity (mOsm/Kg)	553.4 ± 209.8
Urine specific gravity (g/mL)	1.0 ± 0.0
Sodium (mmol/L)	91.6 ± 57.0

g: grams; dL: deciliter; fL: femtoliter; U: units; μL: microliter; mmol: millimol; L: liter; mg: milligrams; μmol: micromole; mOsm: milliosmoles; Kg: kilogram; g: grams; mL: milliliter; ALT: Alanine Transaminase; AST: Aspartate Transaminase; CRP: C-reactive protein. * urine/creatinine ratio > 45 mg/dL and ** a serum osmolarity > 295 mmol/L were used to define dehydration.
